# Rare Earth Elements in the Soil–Grape–Wine System: Opportunities and Limitations for Geographical Origin Authentication

**DOI:** 10.3390/molecules31142437

**Published:** 2026-07-11

**Authors:** Aleksey Abakumov, Zaual Temerdashev, Evgeniy Gipich, Olga Scheludko

**Affiliations:** 1 Analytical Chemistry Department, Faculty of Chemistry and High Technologies, Kuban State University, Krasnodar 350040, Russia; temza@kubsu.ru (Z.T.); jon.gipich@mail.ru (E.G.); 2 North Caucasian Federal Research Center of Horticulture, Viticulture, Wine–Making, Krasnodar 350072, Russia

**Keywords:** elemental “image”, geographical origin, ICP-MS, chemometrics, foods analysis

## Abstract

To establish the relationship between the composition of wine and its regional origin, the most stable chemical parameters are selected because their variation is easiest to trace. Together with “classical” micro- and macroelements, rare earth elements (REEs) can be used for such purposes. However, the relationship between REE content in wine and its regional origin has not been systematically studied. This study examines the migration of REEs in the “soil-grape-wine” system to identify the relationship along the entire chain and establish the geographical origin of grapes and wine. The research was carried out on grapes and wines from the Chardonnay and Cabernet Sauvignon varieties, as well as soil samples selected from different vineyards. The concentrations of La, Ce, Pr, Nd, Sm, Eu, Gd, Tb, Dy, Ho, Er, Tm, Yb and Lu in the studied samples were determined using ICP-MS. It was established that the studied soil samples are characterized by relatively low REE concentrations. Low transfer of REEs from soil to grapes was observed because the soils are rich in clay minerals that firmly retain these elements. Statistically significant differences in the REE content of grapes from different regions were detected (*p* < 0.05). Multidimensional analysis methods allowed us to group grape samples into clusters based on varietal and regional origin. Discriminant analysis showed the possibility of using the concentration of REEs as markers of the geographical origin of grapes. Of all the REEs, only La and Ce are reliably established in all wines. When determining the geographical origin of wine, the use of REE content is limited due to their very low concentrations in wine. In this regard, it has been proposed to use them in combination with macro- and microelements, which improves the classification performance of the model.

## 1. Introduction

Rare earth elements (REEs) are an important tool for identifying various objects and are widely used as geochemical markers [1,2,3]. Their presence in soils is primarily due to the processes of soil formation. Carbonatites are the main natural source of REEs in soils [4,5,6,7,8]. REEs are absorbed by plants from the soil through their root systems and is distributed to different parts of the plant in the following order: roots > leaves > stems > fruits [9,10]. The concentration of REEs in a plant correlates with their content in the soil, which makes it possible to establish traceability and use them as terroir markers [11].

The REE content in wine is quite low, ranging from 0.1 to 20 µg/L [12,13,14]. However, it is actively used as a marker to establish the geographical origin of the drink worldwide [13,15,16,17,18,19,20,21,22]. It should be noted that their use as independent markers is quite limited. As a rule, when identifying and differentiating wines, the REE content is of interest only in combination with other elements. For example, in the study of Chinese wine in [23], Y and Ce were the only significant markers among REEs. In other studies [24,25], the REE content was considered as a marker but were not significant. Moreover, there is direct evidence of their insufficient independence as markers by the authors of [13]. They were able to identify only seven out of 12 wine-growing regions based on REE concentrations, which required the use of data on other elements. Despite their informative value, it can be concluded that the possibilities and limitations of using REEs as markers of geographical origin in wine require clarification [26].

The main disadvantage of most studies is the methodological fragmentation. Researchers mainly analyze commercial wines, leaving the processes occurring in the “soil-grape-wine” chain unexplored. Only a few studies attempt to evaluate the entire system comprehensively. For example, in [27], it was shown that the transition of REEs from soil to wine can be traced, but the treatment of wine with bentonite significantly changes the REE profile, disrupting its connection with terroir. This study differs from previous ones in its comprehensive approach to assessing the distribution of REEs in the soil–grapes–wine system. However, it has some shortcomings in data processing related to the assessment of commonly accepted indices of element transfer.

The authors [28,29] showed that winemaking technology is the key limiting factor in reducing the reliability of REEs content as markers, as it can significantly change the initial elemental profile inherent in grapes. The fining stage has the strongest effect. In particular, treatment of wine with bentonite clay can lead to an increase in the concentration of rare earth elements due to their leaching from the clay structure. This was shown using Cabernet Sauvignon, Merlot, and Moldavian wine samples as examples [14,30]. Thus, the use of concentrations of REEs as markers of wine origin is complicated by their low concentrations and the strong influence of production technology. Currently, few studies have been published on the traceability of REEs through the entire “soil-grape-wine” system. Existing research focuses either on agrochemistry (the “soil-grape” transition) or on oenology (the effect of winemaking technology). To reliably use REE content as a marker of origin, an integrated approach is required to trace its migration throughout all stages of wine production.

The purpose of this work is to study the distribution of rare earth elements in the soil–grape–wine system and to assess the possibility of using them as markers of geographical origin of grapes and wine.

## 2. Results and Discussion

### 2.1. Elemental Composition of Vineyard Soils

The elemental composition of soils and the availability of elements depend on their mineralogical composition. To establish the mineralogical profile and determine the main phases present in soils, X-ray phase analysis was carried out [31,32]. It was found that the vineyard soils for Cabernet Sauvignon and Chardonnay grapes were almost identical (Figure S1). The main mineral in these soils is quartz (31–35%), with other phases represented by illite, nontronite, muscovite, and albite. Anapa soils are classified as silicate–carbonate in their mineralogical composition. Their quartz content is 25%, and they also contain illite, nontronite, albite, and dolomite. Furthermore, soils from Anapa’s Cabernet Sauvignon vineyards are characterized by high contents of zeolite and calcite (Figure S2). The greatest differences in mineralogical composition were observed in samples of carbonate soils from Gostagayevskaya. For this area, calcite dominated the entire soil profile in Cabernet Sauvignon vineyards and its content increased with depth. As for the soils of the Chardonnay vineyards, calcite was found only at a depth of 140–160 cm (Figure S3). As can be seen, despite the proximity of the territories, the differences in the composition of the soils of the vineyards where the two grape varieties are grown are noticeable. The other phases in these soils were illite, muscovite, nontronite, and albite. Mineralogical differences among the vineyard soils were the basis for considering these samples as independent study objects.

The REE content in soil samples, grapes, and wines of the Cabernet Sauvignon and Chardonnay varieties is shown in Table 1. As can be seen from the table, Ce is the most abundant REE in soils for all vineyards, followed by La and Nd. The distribution of REE concentrations in the soil samples largely corresponds to the order of their abundances in the Earth’s continental crust: Ce > La > Nd > Sm > Eu > Yb > Tb > Lu. In addition, the distribution of rare earth elements (REEs) in soils and grapes follows the Oddo–Harkins rule [33]. According to the rule, even-numbered nuclides are more common than their odd-numbered neighbors. Therefore, the distribution has a sawtooth profile with decreasing prevalence (Table 1).

**Figure 1:**
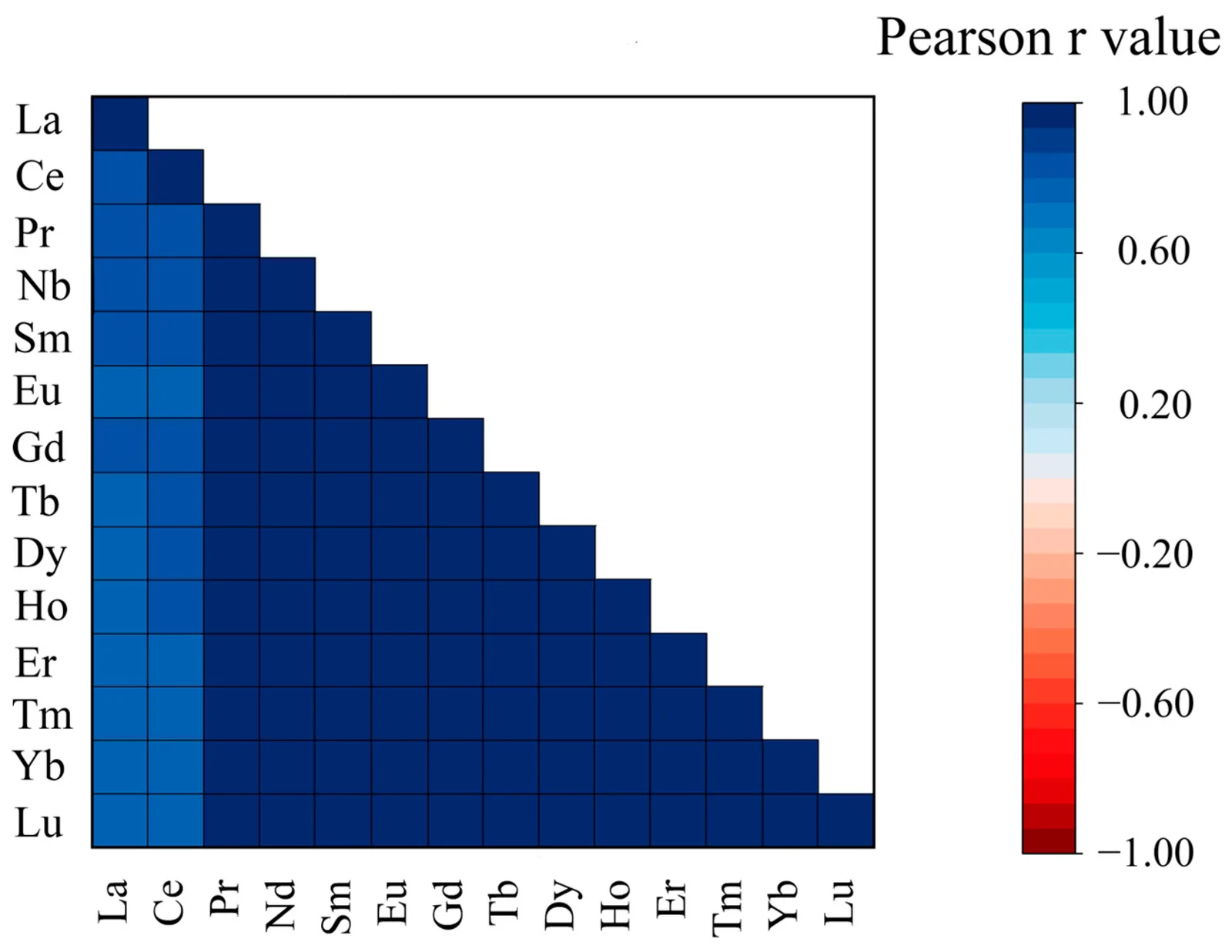
Correlation matrix of rare earth elements in soils.

The concentrations of rare earth elements in soils show a strong correlation (Figure 1). The same patterns have been noted by other authors [34]. Some of them [35] successfully use concentrations of individual rare earth elements to predict their total concentration in soils.

**Figure 2:**
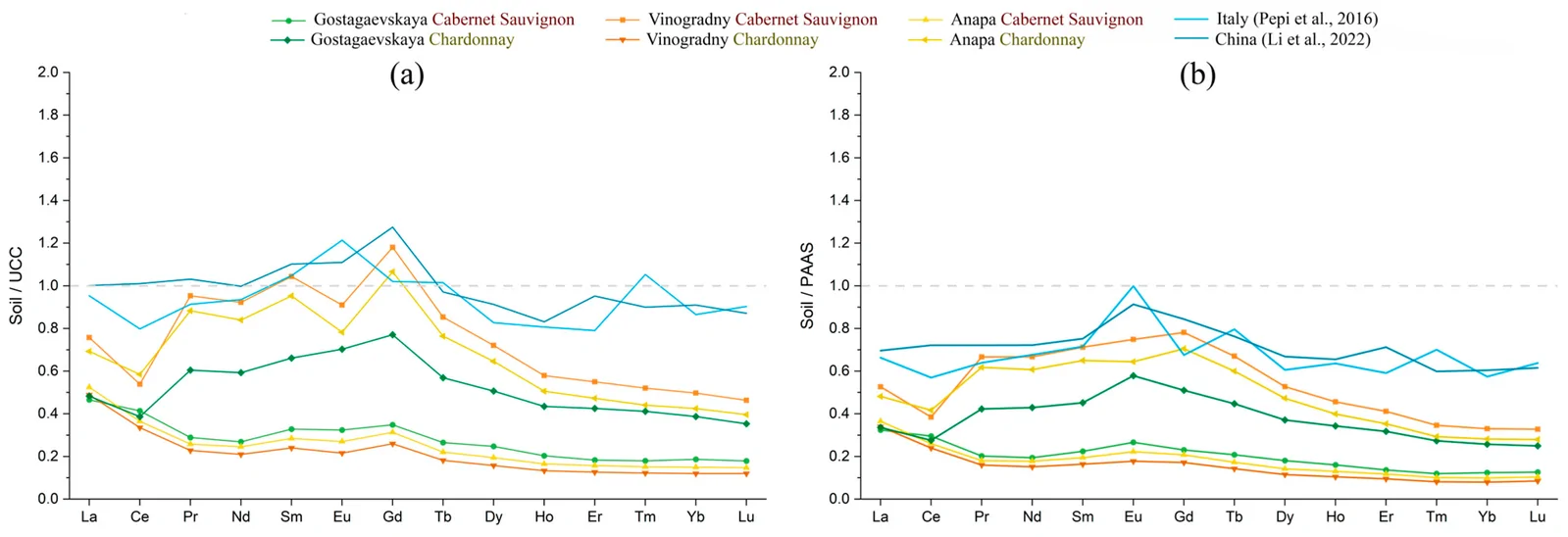
Concentrations of rare earth elements in soil samples, normalized relative to those in: (**a**) the upper continental crust; (**b**) Post-Archean Australian Shale [12,39].

The highest total REE concentration was observed in the soils from Vinogradny (106 mg/kg). These soils are characterized by clay texture and formed from hard limestone [31,32,36]. The lowest total REE concentration was observed in the soils from Anapa (52 mg/kg). To assess the enrichment of REEs in soils, their concentrations were normalized to values in the upper continental crust (UCC) (Figure 2a) [37] and Post-Archean Australian Shale (PAAS) (Figure 2b) [38]. The REE distribution models show that REE concentrations are depleted in all studied soils.

**Figure 3:**
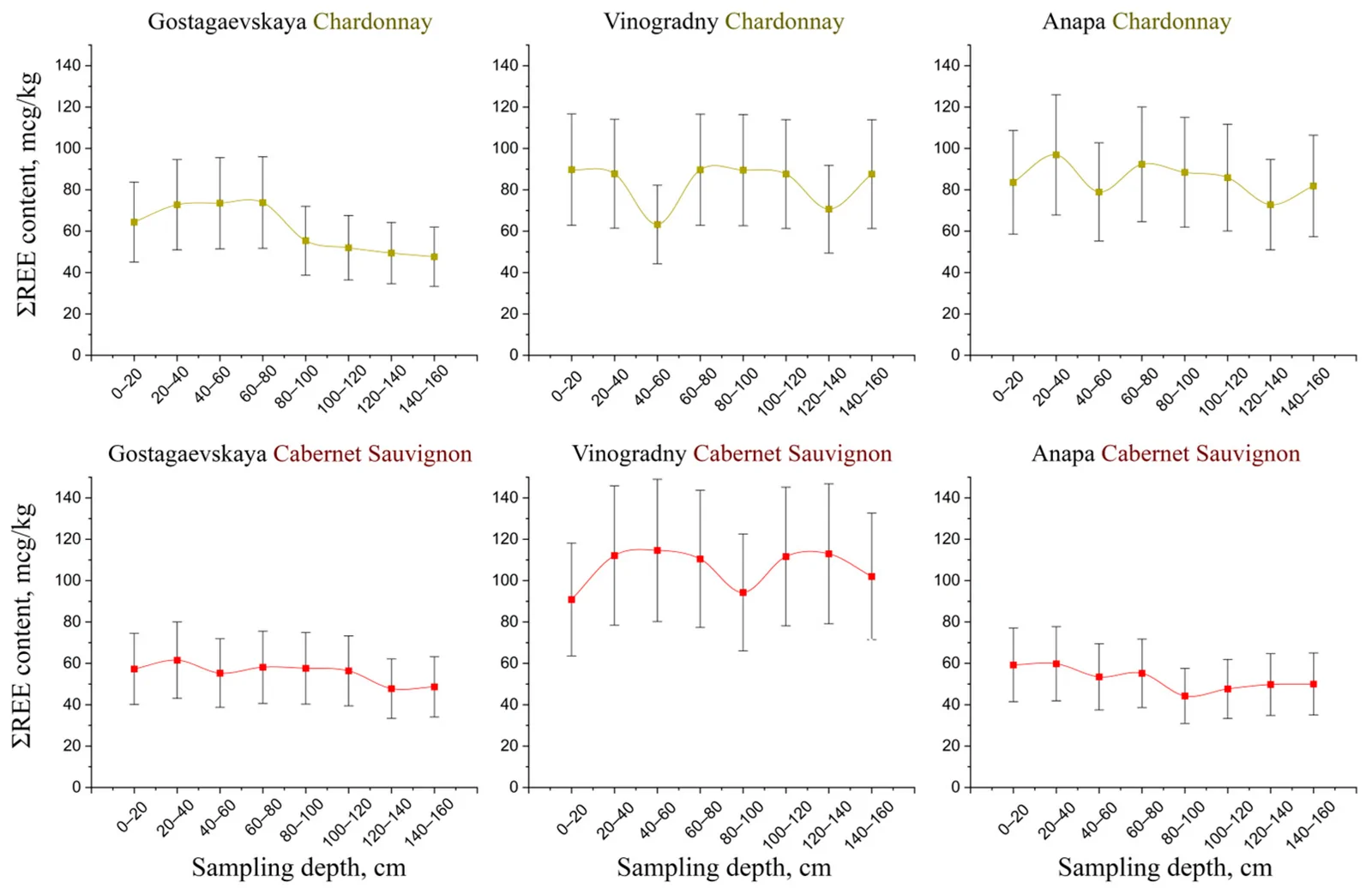
The distribution of ΣREE in the soil profile of vineyards.

Analysis of the REE distribution in the soil profile shows that there are no significant changes in element concentrations as sampling depth increases (Figure 3). This confirms that soil formation processes have virtually no effect on the REE content in soils [33]. Despite the absence of significant changes, lower total REE concentrations were observed in the upper layer of the soil profile for all vineyards (0–20 cm). This is probably due to weathering processes, which significantly affect the behavior of REEs under natural conditions [39].

### 2.2. Elemental Composition of Grapes

Regardless of the region and variety, most of the REE content in grape samples is represented by light REEs, the concentrations of which decrease with the following order: Ce > La > Nd > Pr > Sm > Eu (Table 1). The content of heavy REEs also decreases in the following order: Gd > Dy > Er > Yb > Tb > Ho > Tm > Lu.

According to the results of the one-way ANOVA (*p* < 0.05), statistically significant differences were found among the study areas for each REE. Despite the relatively high total content of REEs in the soil, their content in grapes is much lower (Table 1). This is primarily due to the low mobility of REEs and their uneven distribution in the parts of the plant [9,10]. The mobility of REEs in soil is related to pH, chemical availability, organic matter, fertilizers, redox potential, and texture [40]. In addition, sorption onto clay minerals, phosphates, hydroxides of Fe, Mn and Al, and soil organic matter reduces the solubility and mobility of REEs in soils [41]. The authors of [42] also noted that REEs accumulate differently in different parts of the vine. Their highest concentrations were observed in the roots and leaves, and the lowest in grapes.

### 2.3. Correlations Between the Soil and Grape

To study the uptake of REEs by grapes, the biological absorption coefficient (BAC) was calculated as the ratio of the concentration of an element in grapes to its concentration in the soil (Table 2). BAC provides information on the relative availability of REEs in the soil for uptake by plants [40,43]. The calculated BAC values are shown in Table 2.

The data in Table 2 confirms that there are differences in REE absorption based on both varietal and regional factors. The low BAC of REEs can be explained by the mineralogical composition of the soil. The studied soils of the Anapa region contain clay minerals, including illite and vermiculite [31]. Therefore, the retention of rare earth elements (REEs), especially light REEs, in soils may be associated with the adsorption of these elements by minerals [41]. For all samples, heavy REEs —Tm, Yb and Lu—have the highest BAC.

**Figure 4:**
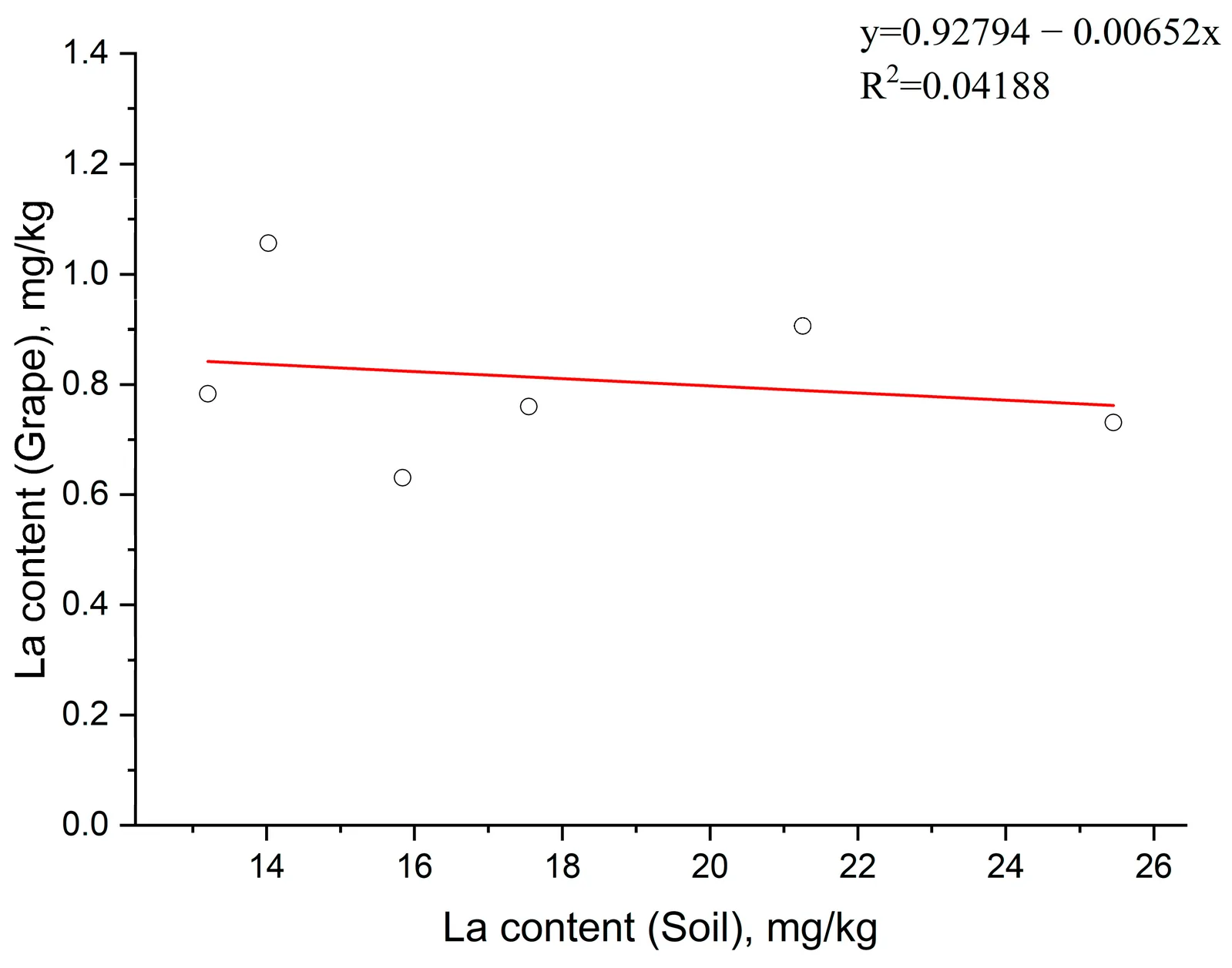
The scatterplot of La content in soil and grape samples.

The different absorption and accumulation of elements in the vine make it difficult to trace elements in the “soil–grape” chain. To identify the correlations between the concentrations of elements in soil and grapes, a Pearson correlation analysis was performed. It has been established that there are no statistically significant correlations between the concentrations of elements in soil and grapes. Additionally, to analyze the correlation between the elemental composition of soil and grapes, a Mantel test was performed. This test allows the establishment of a correlation between two matrices. The test revealed the absence of a correlation between soil and grape composition (premutation *N* = 10,000; correlation R = −0.09105; *p* = 0.5643). A graphical representation of the lack of a relationship between the concentrations of elements in soil and grapes is provided by the scatterplot of La content (Figure 4).

In the context of the terroir concept, the central analytical task is to search for reliable geochemical markers that make it possible to link the elemental composition of wine to the place where the grapes were grown [44]. The ideal marker is considered to be an element whose content in a plant (and, further, in wine) predictably correlates with its concentration in soil. However, in practice, a direct correlation is often not observed. Most researchers [45,46,47] agree that the strongest and most stable positive correlation is characteristic of the “soil-leaf” system. In this case, the direct “soil-berry” relationship, which is of key importance in this case, often turns out to be weak or statistically insignificant [31].

This fact significantly complicates the justification of markers of origin based directly on the composition of the final product. The reason for this discrepancy is that the vine is a complex, dynamically regulated system. During the growing season, the plant’s mineral needs and ways of redistributing them change. It is the physiological control of plant nutrition that explains why the concentration of many elements in grapes is strictly regulated by the needs of the plant, and not only by the total content of the element in the soil [48]. This leads to a weakening of the direct “soil-grape” relationship [49,50].

### 2.4. Establishing the Geographical Origin of Grape

The authors of [13,14,15,16,17,18,19,20,21,22] demonstrated the possibility of using concentrations of REEs as marker elements for varietal and regional classification. Based on this, the data on the REE content in the grape samples was analyzed by discriminant analysis and then graphically illustrated to assess their similarity and difference. The territory of grape growth and its variety was used as a grouping variable (6 groups). The concentrations of REEs in the grape samples were selected as predictors (independent variables). A step-by-step exclusion method was used to select the most informative predictors, which allows for the gradual elimination of redundant predictors from the model based on F-remove statistics. Table 3 shows that discrimination was successfully carried out. This is confirmed by the Wilks’ lambda value, which tends towards zero. Over four steps, Dy, Sm, Ho and Gd were excluded from the model. All remaining predictors in the model are statistically significant at *p* < 0.00000. Cross-validation was performed to assess the quality of the grape discrimination model. The dataset was randomly split into a train (80%) and a test (20%) set, maintaining the same proportions between the groups. The classification accuracy for both sets was 100%.

**Figure 5:**
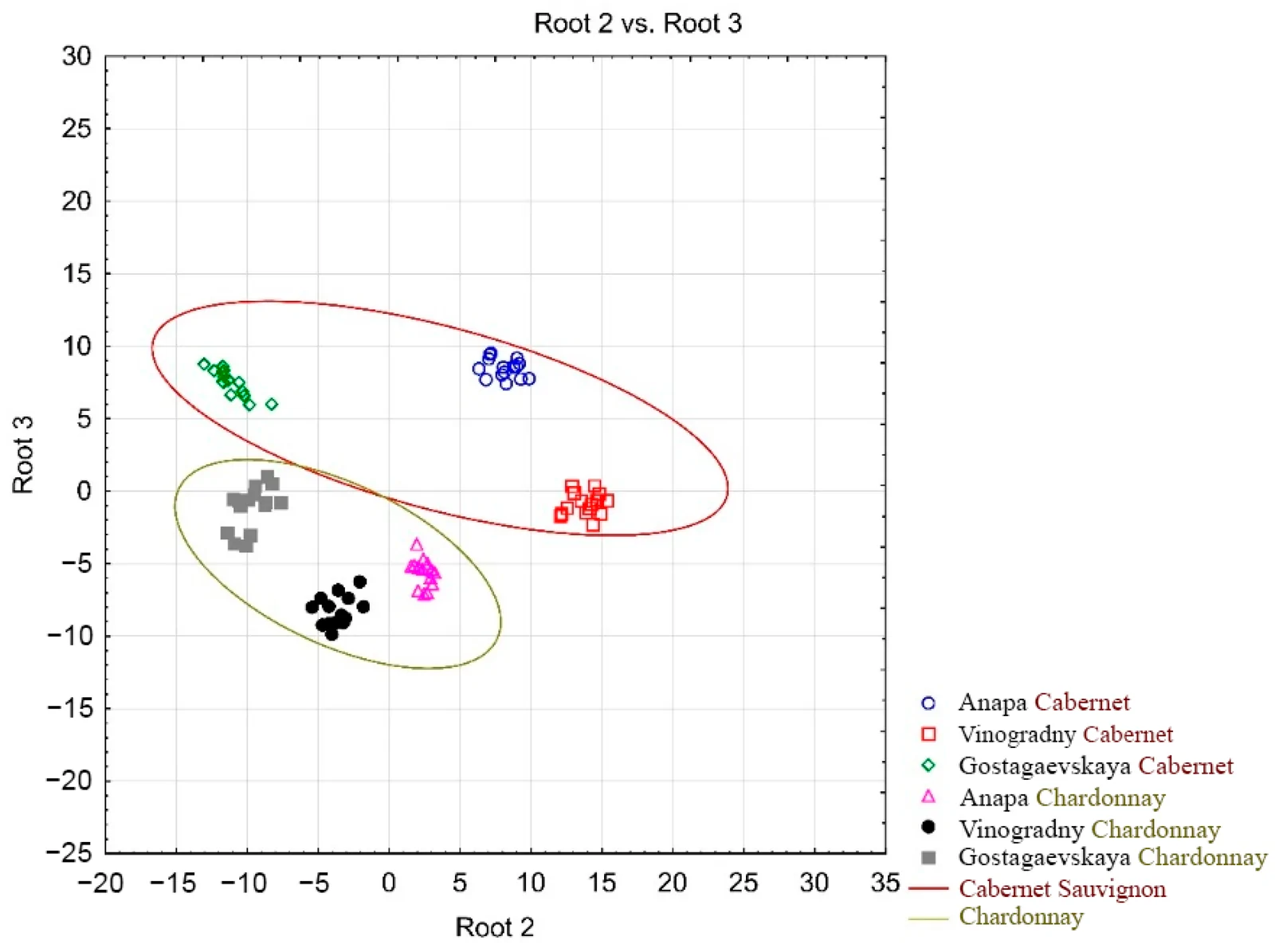
A scatterplot of the canonical values of grape samples constructed using REEs as predictors.

The scatterplot of canonical values allows you to transfer objects from multidimensional space into a space with a smaller dimension—a plane, while preserving the relative distances between objects. The resulting scatterplots of the canonical values of grape samples constructed using REEs as predictors will allow us to judge the uniformity of objects and the degree of similarity or difference between groups by comparing distances. The smaller the distance, the greater the similarity. It can be seen from Figure 5 that grape samples form groups of homogeneous objects—clusters. It should be noted that samples of Cabernet Sauvignon grape samples are located mainly in the upper part of the plane and Chardonnay—in the lower part (highlighted by ellipses). This may indicate the possibility of using REEs as additional predictors for establishing varietal affiliation.

The absence of a statistically significant correlation between the concentrations of REEs in soil and grapes does not exclude the possibility of using them as markers of geographical origin. At the same time, the geographical marker element does not always imply a quantitative transfer of the element from the soil to the plant. Above all, a marker needs reproducibility and regional distinctness of its content in the final product. Despite the fact that the availability of REEs is primarily determined by their sorption by clay minerals and biological barriers of plants, the nature of their transition from soil to plants remains regionally specific. The results obtained suggest that a model based on REE concentrations can be used to determine the regional origin of grapes, even if they are grown in soils that are not enriched with REEs (Figure 5).

### 2.5. Elemental Composition of Wine

Most of the REEs in the wine samples, despite the high sensitivity of the spectrometer detection, were not detected (Table 1). Of all the REEs, only La and Ce were detected in all wines. The transfer of elements from grapes to wine was estimated using the transfer factor (TF), calculated according to the equation proposed by [51]:

(1) $$TF=\frac{C_{wine}}{C_{grape}},$$

*C_wine_* and *C_grape_*—concentrations of elements in wine and grapes, respectively.

Analysis of the data obtained (Table 4) showed that only the red wine samples from Vinogradny and Anapa had La and Ce transfer factors higher than 0.0005. In other wine samples, the REE content was more than 3.5000-fold lower compared to its initial content in grapes. We also noted that the TF value was higher in red Cabernet Sauvignon wine compared to white Chardonnay wine from the same region. Apparently, this is due to differences in the distribution of REEs in different parts of the grape berry and/or differences in production processes for red and white wines [52].

### 2.6. Establishing the Geographical Origin of Wine

The obtained data on the elemental composition of wines shows that due to the low REE content, it is almost impossible to accurately assess the regional origin of the wine. Using only REEs as markers for wines produced from grapes grown in soils with low levels of REEs can lead to incorrect identification. Therefore, it would be advisable to select more specific marker elements from macro- and microelements, or to combine them with REEs [13,14,15]. To establish the geographical origin of the wine samples, 21 micro- (Al, As, Ba, Be, Cd, Co, Cs, Cu, Fe, Li, Mn, Mo, Ni, Pb, Rb, Sr, Ti, V, W, Zn, Zr) and 4 macroelements (Na, Mg, Ca, K) with REEs were used as predictors (Table S1). The results of discriminant analysis of wine samples showed that Ce is one of the significant predictors (Table 5). At the same time, Table 5 shows that Ce makes the smallest contribution (the highest partial lambda) and Fe makes the largest contribution (the lowest partial lambda) to the discriminant model.

**Figure 6:**
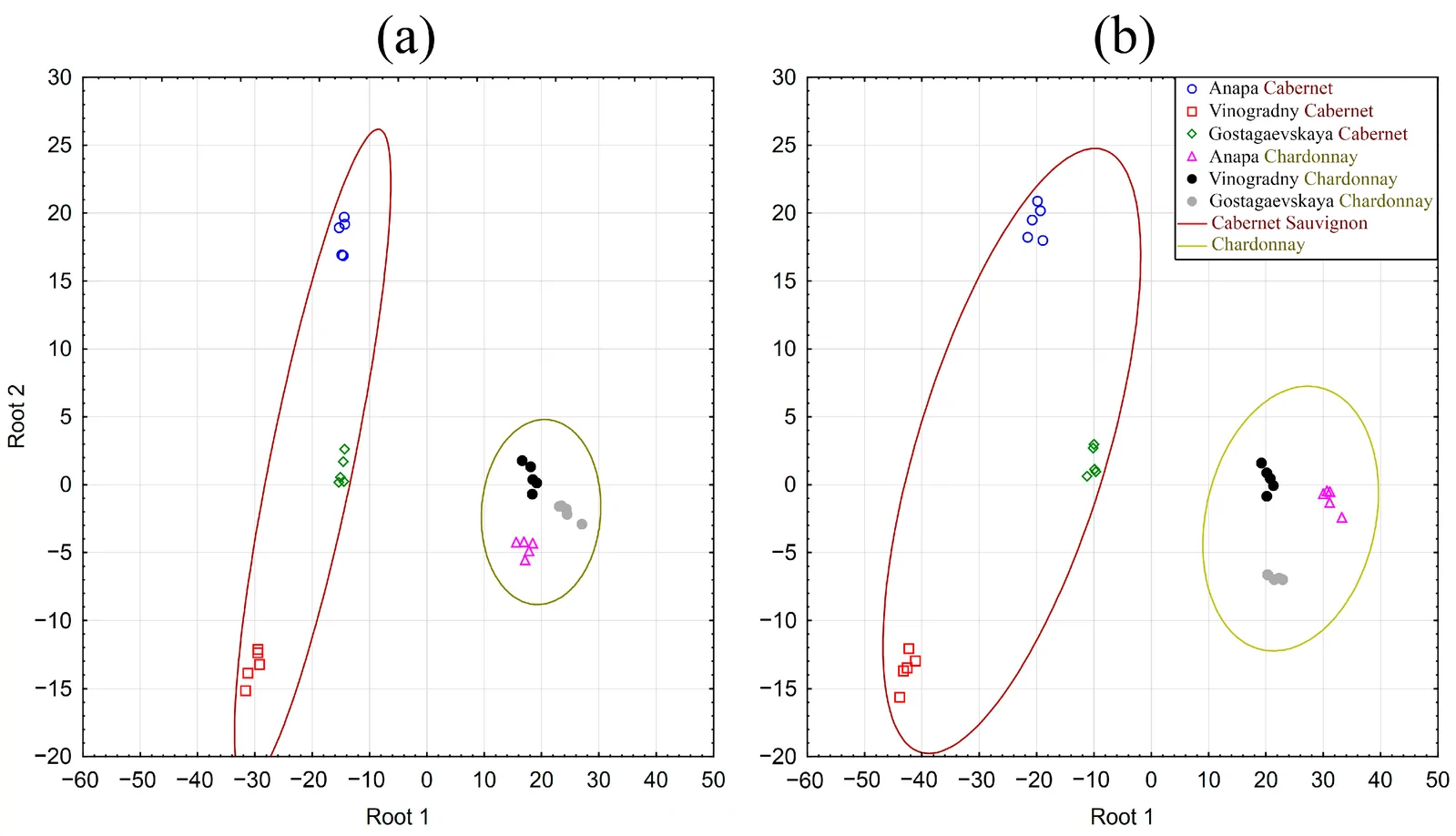
Scatterplots of the canonical values of wine samples, constructed using concentrations of macro- and microelements (**a**), and the additional inclusion of Ce as a predictor (**b**).

Wine samples form highly distinct clusters, as shown in the scatterplot of canonical values (Figure 6b). At the same time, it should be noted that samples of different varieties are located in different parts of the plane, which indicates a tendency to form varietal clusters. To compare, a non-REE discrimination model was created, in which the initial predictors were only the macro- and microelements (Figure 6a). In the absence of La and Ce in the dataset, Na contributes the most to discrimination, and Zn—the least (Table 5). Despite some differences in the predictors, the resulting scatterplots are quite similar. It is worth noting that, with the addition of La and Ce predictors (Figure 6b), the distances between clusters increase, which indicates an improvement in the discriminatory performance of the model. To assess the quality of wine discrimination models, cross-validation is not practical due to the small number of observations in each group (*N* = 5). Thus, the entire dataset was used to build models and the classification accuracy of both models was 100%. Mahalanobis distances were used to estimate the potential for identification properties of the models. Squared Mahalanobis distances serve as a measures of the distances between points and cluster centroids. It was found that in the REE model, Mahalanobis distances were greater between different groups (Table S2) compared to the non-REE model (Table S3). Thus, an increase in intergroup distances indicates discriminatory performance of the model.

## 3. Materials and Methods

### 3.1. Objects of Research

The study objects were samples of Cabernet Sauvignon and Chardonnay grapes taken from the vineyards in the Anapa region, as well as the soil in which the grapes were grown. Soil samples were collected using the “envelope” method during the berry ripening period. In our previous studies [31,32], we determined the mineralogical composition of the studied soils. Despite their proximity (the distance between the vineyards in one area was at least 100 m), differences in the mineralogical composition of the soils from the Cabernet Sauvignon and Chardonnay vineyards were noticeable. Thus, mineralogical differences served as the basis for considering these samples independent study objects. The information about the studied vineyards is presented in Table S4. Grapes were harvested when they reached their technical maturity. The processing of grapes and the production of young wines was carried out in accordance with general rules and technological instructions for wine production. The wines were produced from grapes harvested in 2022.

The young Cabernet Sauvignon wine was prepared by fermenting the must with active dry yeast (Lallemand Lalvin EC 1118, 0.3 g/hL). The fermenting must was mixed 3–4 times per day while maintaining the temperature at 28–32 °C for 7 days. During fermentation, “Ultrasulph C” was added at a concentration of 160 g/t (equivalent to 100 mg/L of SO_2_). After fermentation, the must was pressed using a “Busher Vaslin” (Rivesaltes, France), followed by separation of the wine. Upon completion of sugar fermentation and separation of the yeast sediment, young wine was treated again with “Ultrasulf C” at a dosage of 50 mg/L of SO_2_.

For the production of young Chardonnay wine, drained and pressed juice was used in an amount of no more than 7 hL per 1 ton of grapes. The juice was sulfitized using “Ultrasulf C” at a rate of 80–150 mg of SO_2_ per kg of processed grapes, and clarified by sedimentation. The clarified juice was separated from the sediment and fed into fermentation tanks where active dry yeast was introduced in the dosages recommended by the manufacturers (Lallemand Lalvin EC 1118, 0.3 g/hL). The fermentation temperature did not exceed 18 °C. After sugar fermentation was complete and yeast had settled, young wine was treated with “Ultrasulf C” again at a rate of 50 mg/L SO_2_. The main physicochemical properties of the Cabernet Sauvignon and Chardonnay wines obtained are presented in Table S5.

### 3.2. Materials and Reagents

Concentrated H_2_O_2_, HF, HCl and HNO_3_ were used in experimental studies (all of high purity, PanReac AppliChem, Darmstadt, Germany). To determine the elemental composition of the samples, multi-element calibration solutions were prepared from single-element standard solutions of Ce, Dy, Er, Eu, Gd, Ho, La, Lu, Nd, Pr, Sm, Tb, Tm and Yb with a concentration of 1000 mg/L (Inorganic Ventures, Christiansburg, VA, USA). The analyzed solutions were prepared using deionized water with a resistivity of 18.2 MΩ·cm^−1^, which was obtained from a DuoPUR (Milestone, Milan, Italy) sub-distillation system.

### 3.3. Elemental Analysis

The elemental analysis was performed using inductively coupled plasma mass spectrometry (ICP-MS) on an iCAP RQ quadrupole mass spectrometer (Thermo Scientific, Waltham, MA, USA) [53]. The determined isotopes of the elements and their limits of detection are summarized in Table 6.

### 3.4. Preparation of Soil Samples for Analysis

To determine the total content (TC) of elements, soil samples were digested using an Ethos 1 microwave digestion system (Milestone, Milan, Italy) using the method described in [49]. For this purpose, 0.50 g of soil and an oxidizing mixture containing 5.0 mL of concentrated HF, 3.0 mL of concentrated HNO_3_, and 1.0 mL of concentrated HCl were placed in an autoclave. To digest the samples, the analyzed mixture was gradually heated to 200 °C for 25 min, and then kept at 200 °C for 5 min in the reaction chamber. To avoid losses of volatile elements, the autoclaves were opened at temperatures below 40 °C; then, the contents of the autoclave were transferred to a 50 mL polypropylene volumetric flask and brought up to the mark with deionized water. ICP-MS analysis of soil samples was carried out using an HF-resistant sample injection system, including a PFA-ST nebulizer (Thermo Scientific, Waltham, MA, USA), a cyclone-type PFA spray chamber (Glass Expansion, Melbourne, Australia), and a 2.0 mm corundum injector (Glass Expansion, Melbourne, Australia).

### 3.5. Preparation of Grape Samples for Analysis

The elements in grapes were quantified according to [54]. The digestion of grapes before analysis was carried out using an Ethos 1 microwave system (Milestone, Italy). The berries were washed with distilled water and dried; then, 2.0 g of grapes was placed in an autoclave. Digestion of the sample was carried out at elevated pressure using an oxidizing mixture consisting of 5.0 mL of concentrated HNO_3_, 1.0 mL of H_2_O_2_, and 4.0 mL of deionized water. The sample digestion program included gradual heating to 200 °C for 10 min (stage 1) and maintaining the reaction chamber at 200 °C for 10 min (stage 2). Autoclaves were opened at temperatures below 40 °C to avoid losses of volatile elements. Then, the content of the autoclave was transferred to a 25 mL flask and brought to the mark with deionized water.

### 3.6. Preparation of Wine Samples for Analysis

The preparation of wines to establish elemental composition consisted of a 15-fold dilution of the samples with 2% HNO_3_ according to [20,55].

### 3.7. Validation of Methods Used

The accuracy of the soil sample preparation procedure was established by analyzing standard soil samples. The sample preparation of grapes and wine samples were evaluated using recovery tests. The assessment of the correctness of the applied methods is presented in Tables S6–S8.

### 3.8. Statistical Analysis

The analysis of the relationships between the elemental composition of soils, grapes and wine was carried out using the STATISTICA (v. 14.0.0.15) and OriginPro (v. 10.2.5.212) software [56]. Correlations between metal concentrations were assessed using the Pearson’s correlation coefficient. When |*r*| ≤ 0.25, the correlation was considered weak; when 0.25 < |*r*| ≤ 0.75, it was considered moderate; and when |*r*| > 0.75, it was considered strong. The correlation between the elemental profiles of soil and grapes was assessed using the Mantel test in Past 5 (v. 5.3) software [57]. To perform the Mantel test, mean elemental concentrations were used and the number of permutations was 10,000. To assess statistically significant differences among the samples, a one-way ANOVA (*p* < 0.05), followed by Tukey’s HSD post hoc test (*p* < 0.05), was used. The possibility of using REEs as markers of geographical origin was assessed using discriminant analysis (“step-by-step” exclusion method).

## 4. Conclusions

The opportunities and limitations of using REE concentrations as markers for the geographical origin of grapes and wine are explored using the example of the established relationship between soil, grapes, and wine. The distribution of REE concentrations in the studied soils corresponds to the natural order (Ce > La > Nd, etc.). At the same time, their absolute concentrations were significantly lower than the average values for continental crust.

Statistically significant (one-way ANOVA, *p* < 0.05) differences in REE content were observed in samples of grape varieties from different regions with different mineralogical compositions of soils. Multivariate analysis methods confirm these differences, allowing samples to be grouped into clusters based on their origin. The possibility of using REE concentrations as markers of grape origin is demonstrated.

The use of REE concentrations as markers for wines from the Anapa region is difficult due to the low content of REEs (REE/UCC < 1) in the soils of the studied vineyards. The low coefficient of biological absorption, due to sorption by clay minerals and their weak accumulation in grapes, results in concentrations of REEs in grapes being much lower than those in soils. The consequence of this is that most REEs were not detected in young wines, which fundamentally limits the independent use of REE profiles to determine the origin of wine, and only La and Ce are present in all samples of young wines studied.

It has been shown that REE concentrations (La and Ce) can only be used as markers for the origin of wines in combination with micro- and macroelements. This significantly improves the identification performance of the discriminant model compared to non-REE models. We note that the patterns obtained were based on a dataset with limited geographical coverage from a single region (Anapa region, Russia). Further research will be required to establish these patterns in vineyards in other wine-growing regions.

## Figures and Tables

**Table 1:** REE content in soil, grape and wine samples (mean ± total uncertainty; *p* = 0.95).

Element	Vinograny Cabernet Sauvignon			Vinograny Chardonnay		
	Soil, mg/kg (*n* = 3)	Grape, mg/kg (*n* = 15)	Wine, µg/L (*n* = 5)	Soil, mg/kg (*n* = 3)	Grape, mg/kg (*n* = 15)	Wine, µg/L (*n* = 5)
La	23 ± 7	0.73 ± 0.15	0.57 ± 0.06	19 ± 6	0.78 ± 0.16	0.26 ± 0.03
Ce	34 ± 10	1.5 ± 0.3	1.0 ± 0.1	28 ± 8	1.6 ± 0.3	0.216 ± 0.022
Pr	6.8 ± 2.0	0.18 ± 0.04	0.65 ± 0.06	5.1 ± 1.5	0.20 ± 0.04	<LOQ
Nd	25 ± 7.5	0.74 ± 0.15	<LOQ	19 ± 6	0.76 ± 0.15	<LOQ
Sm	4.9 ± 1.5	0.21 ± 0.04	<LOQ	3.7 ± 1.1	0.21 ± 0.04	<LOQ
Eu	0.91 ± 0.27	0.080 ± 0.016	<LOQ	0.68 ± 0.20	0.140 ± 0.028	<LOQ
Gd	4.7 ± 1.4	0.210 ± 0.042	0.050 ± 0.005	3.6 ± 1.1	0.25 ± 0.05	0.060 ± 0.006
Tb	0.60 ± 0.18	0.030 ± 0.006	<LOQ	0.45 ± 0.14	0.060 ± 0.012	<LOQ
Dy	2.8 ± 0.8	0.090 ± 0.018	<LOQ	2.1 ± 0.6	0.10 ± 0.02	<LOQ
Ho	0.48 ± 0.14	0.030 ± 0.006	<LOQ	0.36 ± 0.11	0.040 ± 0.008	<LOQ
Er	1.3 ± 0.40	0.030 ± 0.006	<LOQ	0.90 ± 0.27	0.060 ± 0.012	<LOQ
Tm	0.16 ± 0.05	0.050 ± 0.01	<LOQ	0.12 ± 0.04	0.05 ± 0.01	<LOQ
Yb	1.0 ± 0.3	0.070 ± 0.014	<LOQ	0.73 ± 0.22	0.10 ± 0.02	0.031 ± 0.003
Lu	0.14 ± 0.04	0.030 ± 0.006	<LOQ	0.1 ± 0.03	0.04 ± 0.008	<LOQ
Total REE	106	4.1	2.266	83	4.5	0.567
**Element**	**Gostagaevskaya Cabernet Sauvignon**			**Gostagaevskaya Chardonnay**		
	**Soil, mg/kg** **(*n* = 3)**	**Grape, mg/kg** **(*n* = 15)**	**Wine, µg/L** **(*n* = 5)**	**Soil, mg/kg** **(*n* = 3)**	**Grape, mg/kg** **(*n* = 15)**	**Wine, µg/L** **(*n* = 5)**
La	14 ± 4	1.1 ± 0.2	0.243 ± 0.024	18 ± 5	0.91 ± 0.18	0.150 ± 0.015
Ce	26 ± 8	2.0 ± 0.4	0.48 ± 0.05	30 ± 9	1.5 ± 0.3	0.20 ± 0.02
Pr	2.1 ± 0.6	0.22 ± 0.04	<LOQ	2.0 ± 0.6	0.230 ± 0.046	<LOQ
Nd	7.3 ± 2.2	1.1 ± 0.2	<LOQ	6.8 ± 2.0	0.730 ± 0.150	<LOQ
Sm	1.5 ± 0.5	0.20 ± 0.04	<LOQ	1.4 ± 0.4	0.190 ± 0.038	<LOQ
Eu	0.32 ± 0.10	0.10 ± 0.02	<LOQ	0.29 ± 0.09	0.120 ± 0.024	<LOQ
Gd	1.4 ± 0.4	0.180 ± 0.036	<LOQ	1.3 ± 0.4	0.190 ± 0.038	<LOQ
Tb	0.19 ± 0.06	0.040 ± 0.008	<LOQ	0.17 ± 0.05	0.070 ± 0.014	<LOQ
Dy	1.0 ± 0.3	0.160 ± 0.032	<LOQ	0.87 ± 0.26	0.080 ± 0.016	<LOQ
Ho	0.17 ± 0.05	0.030 ± 0.006	<LOQ	0.15 ± 0.045	0.040 ± 0.008	<LOQ
Er	0.42 ± 0.1	0.080 ± 0.016	<LOQ	0.40 ± 0.12	0.10 ± 0.02	<LOQ
Tm	0.05 ± 0.015	0.030 ± 0.006	<LOQ	0.05 ± 0.015	0.030 ± 0.006	<LOQ
Yb	0.37 ± 0.11	0.120 ± 0.024	<LOQ	0.35 ± 0.11	0.090 ± 0.018	0.043 ± 0.004
Lu	0.06 ± 0.018	0.020 ± 0.004	<LOQ	0.05 ± 0.015	0.05 ± 0.01	<LOQ
Total REE	56	5.4	0.723	61	4.5	0.393
**Element**	**Anapa Cabernet Sauvignon**			**Anapa Chardonnay**		
	**Soil, mg/kg** **(*n* = 3)**	**Grape, mg/kg** **(*n* = 15)**	**Wine, µg/L** **(*n* = 5)**	**Soil, mg/kg** **(*n* = 3)**	**Grape, mg/kg** **(*n* = 15)**	**Wine, µg/L** **(*n* = 5)**
La	16 ± 5	0.63 ± 0.13	0.450 ± 0.045	19 ± 6	0.76 ± 0.15	0.100 ± 0.010
Ce	23 ± 7	1.2 ± 0.2	0.656 ± 0.066	28 ± 8	1.4 ± 0.3	0.140 ± 0.014
Pr	1.8 ± 0.54	0.17 ± 0.03	<LOQ	5.3 ± 1.6	0.18 ± 0.04	<LOQ
Nd	6.6 ± 2.0	0.61 ± 0.12	<LOQ	19 ± 6	0.59 ± 0.12	<LOQ
Sm	1.3 ± 0.4	0.17 ± 0.034	<LOQ	3.8 ± 1.1	0.17 ± 0.034	<LOQ
Eu	0.27 ± 0.08	0.04 ± 0.008	<LOQ	0.75 ± 0.23	0.07 ± 0.014	<LOQ
Gd	1.3 ± 0.4	0.10 ± 0.02	<LOQ	3.6 ± 1.1	0.20 ± 0.04	<LOQ
Tb	0.15 ± 0.045	0.030 ± 0.006	<LOQ	0.46 ± 0.14	0.06 ± 0.012	<LOQ
Dy	0.76 ± 0.23	0.12 ± 0.02	<LOQ	2.2 ± 0.66	0.08 ± 0.016	<LOQ
Ho	0.14 ± 0.042	0.030 ± 0.006	<LOQ	0.39 ± 0.12	0.04 ± 0.008	<LOQ
Er	0.36 ± 0.108	0.110 ± 0.022	<LOQ	1.0 ± 0.3	0.09 ± 0.018	<LOQ
Tm	0.05 ± 0.015	0.030 ± 0.006	<LOQ	0.13 ± 0.039	0.07 ± 0.014	<LOQ
Yb	0.30 ± 0.09	0.10 ± 0.02	0.118 ± 0.012	0.83 ± 0.25	0.14 ± 0.028	<LOQ
Lu	0.05 ± 0.015	0.04 ± 0.008	<LOQ	0.12 ± 0.04	0.040 ± 0.008	<LOQ
Total REE	52	3.4	1.222	85	4.0	0.241

**Table 2:** The calculated values of biological absorption coefficients.

Element	Vinogradny		Gostagaevskaya		Anapa	
	Cabernet Sauvignon	Chardonnay	Cabernet Sauvignon	Chardonnay	Cabernet Sauvignon	Chardonnay
La	0.031 ^d^	0.042 ^c^	0.073 ^a^	0.051 ^b^	0.039 ^cd^	0.040 ^c^
Ce	0.044 ^c^	0.057 ^b^	0.078 ^a^	0.052 ^b^	0.051 ^b^	0.049 ^b^
Pr	0.027 ^d^	0.04 ^c^	0.106 ^ab^	0.118 ^a^	0.091 ^b^	0.034 ^cd^
Nd	0.030 ^c^	0.041 ^c^	0.152 ^a^	0.107 ^b^	0.092 ^b^	0.030 ^c^
Sm	0.040 ^c^	0.056 ^b^	0.127 ^a^	0.135 ^a^	0.127 ^a^	0.045 ^bc^
Eu	0.087 ^d^	0.210 ^c^	0.315 ^b^	0.415 ^a^	0.155 ^c^	0.094 ^d^
Gd	0.045 ^c^	0.070 ^b^	0.131 ^a^	0.142 ^a^	0.077 ^b^	0.054 ^c^
Tb	0.048 ^d^	0.141 ^c^	0.224 ^b^	0.406 ^a^	0.213 ^b^	0.137 ^c^
Dy	0.033 ^d^	0.049 ^d^	0.169 ^a^	0.092 ^c^	0.126 ^b^	0.035 ^d^
Ho	0.053 ^d^	0.115 ^c^	0.184 ^b^	0.268 ^a^	0.215 ^b^	0.103 ^c^
Er	0.021 ^e^	0.061 ^d^	0.182 ^b^	0.263 ^a^	0.308 ^a^	0.092 ^c^
Tm	0.294 ^d^	0.454 ^c^	0.589 ^ab^	0.671 ^a^	0.552 ^b^	0.548 ^b^
Yb	0.069 ^d^	0.132 ^c^	0.313 ^ab^	0.249 ^b^	0.343 ^a^	0.171 ^c^
Lu	0.218 ^d^	0.356 ^bc^	0.426 ^b^	0.960 ^a^	0.840 ^a^	0.314 ^c^

Note: different letters in the same row indicate significant differences at *p* < 0.05 (Tukey’s HSD test).

**Table 3:** The results of discriminant analysis of grape samples.

*N* = 90	Step 4, *N* of Vars in Model: 10; Wilks’ Lambda: 0.00000 Approx. F (50.345) = 380.76 *p* < 0.00000					
	Wilks’ Lambda	Partial Lambda	F-Remove (5.19)	*p*-Value	Toler.	1-Toler. (R-sqr.)
La	0.000000	0.1789	68.804	0.000000	0.4498	0.5501
Ce	0.000000	0.4290	19.961	0.000000	0.5665	0.4334
Pr	0.000000	0.5348	13.046	0.000000	0.3797	0.6202
Nd	0.000000	0.3779	24.691	0.000000	0.7483	0.2516
Eu	0.000000	0.3058	34.037	0.000000	0.5684	0.4315
Tb	0.000000	0.3360	29.636	0.000000	0.5625	0.4374
Er	0.000000	0.1630	76.970	0.000000	0.6624	0.3374
Tm	0.000000	0.3787	24.605	0.000000	0.7165	0.2834
Yb	0.000000	0.1786	68.967	0.000000	0.6174	0.3825
Lu	0.000000	0.4116	21.442	0.000000	0.6839	0.3160

**Table 4:** The calculated values of transfer factors.

Element	Vinogradny		Gostagaevskaya		Anapa	
	Cabernet Sauvignon	Chardonnay	Cabernet Sauvignon	Chardonnay	Cabernet Sauvignon	Chardonnay
La	0.00076 ^a^	0.00030 ^c^	0.00021 ^d^	0.00016 ^d^	0.00068 ^b^	0.00013 ^d^
Ce	0.00069 ^a^	0.00014 ^d^	0.00024 ^c^	0.00013 ^d^	0.00055 ^b^	0.00011 ^d^
Pr	0.00347 ^a^	— *	—	—	—	—
Nd	—	—	—	—	—	—
Sm	—	—	—	—	—	—
Eu	—	—	—	—	—	—
Gd	0.00023 ^a^	0.00023 ^a^	—	—	—	—
Tb	—	—	—	—	—	—
Dy	—	—	—	—	—	—
Ho	—	—	—	—	—	—
Er	—	—	—	—	—	—
Tm	—	—	—	—	—	—
Yb	—	0.00033 ^c^	—	0.00047 ^b^	0.00128 ^a^	—
Lu	—	—	—	—	—	—

Note: different letters in the same row indicate significant differences at *p* < 0.05 (Tukey’s HSD test); — *—equal to zero due to the absence of the element in the wine.

**Table 5:** Results of discriminant analysis of the wine samples.

**ONLY** **Micro- and** **Macroelements**	***N* = 30**	**Step 15, *N* of Vars in Model: 5;** **Wilks’ Lambda: 0.00000 Approx. F (25.75) = 654.34 *p* < 0.0000**					
		**Wilks’** **Lambda**	**Partial** **Lambda**	**F-Remove** **(5.19)**	***p*-Value**	**Toler.**	**1-Toler.** **(R-sqr.)**
	Na	0.000000	0.0145	271.78	0.000000	0.8977	0.1022
	Cd	0.000000	0.0147	267.24	0.000000	0.8803	0.1196
	Fe	0.000000	0.0280	138.41	0.000000	0.9456	0.0543
	Cu	0.000000	0.0513	73.91	0.000000	0.8485	0.1514
	Zn	0.000000	0.1036	34.59	0.000000	0.8329	0.1670
**Microelements,** **Macroelements, +La, Ce**	***N* = 30**	**Step 16, *N* of vars in model: 6;** **Wilks’ Lambda: 0.00000 Approx. F (30.78) = 459.63 *p* < 0.0000**					
		**Wilks’** **Lambda**	**Partial** **Lambda**	**F-Remove** **(5.19)**	***p*-Value**	**Toler.**	**1-Toler.** **(R-sqr.)**
	Fe	0.000000	0.0192	193.71	0.000000	0.7430	0.2569
	Cd	0.000000	0.0213	173.98	0.000000	0.8888	0.1111
	Cu	0.000000	0.0517	69.60	0.000000	0.7143	0.2856
	Na	0.000000	0.0878	39.43	0.000000	0.7797	0.2202
	Mg	0.000000	0.2273	12.91	0.000015	0.7672	0.2327
	Ce	0.000000	0.2587	10.88	0.000047	0.6319	0.3680

**Table 6:** *m*/*z* of the measured elements and their limits of quantification.

Element	*m*/*z*	LOQ, µg/L	Element	*m*/*z*	LOQ, µg/L
La	139	0.001	Tb	159	0.005
Ce	140	0.001	Dy	163	0.003
Pr	141	0.011	Ho	165	0.006
Nd	146	0.014	Er	166	0.003
Sm	147	0.006	Tm	169	0.002
Eu	153	0.003	Yb	172	0.002
Gd	157	0.002	Lu	175	0.002

## Data Availability

All data are included in the article.

## Supplementary Materials

The following supporting information can be downloaded at: https://www.mdpi.com/1420-3049/31/14/2437/s1. Figure S1: Diffractograms of soil samples from Cabernet Sauvignon and Chardonnay vineyards located in Vinogradny; Figure S2: Diffractograms of soil samples from Cabernet Sauvignon and Chardonnay vineyards located in Anapa; Figure S3: Diffractograms of soil samples from Cabernet Sauvignon and Chardonnay vineyards located in Gostagaevskaya; Table S1: The concentrations of elements in wines; Table S2: Squared Mahalanobis Distances from Group Centroids in the REE model; Table S3: Squared Mahalanobis Distances from Group Centroids in the non-REE model; Table S4: Information about the studied vineyards; Table S5: Physicochemical properties of Cabernet Sauvignon and Chardonnay wines; Table S6: Determination of REEs concentrations in CRM Soil (NCS DC 77302); Table S7: Determination of REE concentrations in grapes; Table S8: Determination of REEs concentrations in wines.
