# Supplementary material for: Rare Earth Elements in the Soil–Grape–Wine System: Opportunities and Limitations for Geographical Origin Authentication
Source: Molecules. 2026 Jul 11;31(14):2437. doi: 10.3390/molecules31142437 (PMC13415107; doi:10.3390/molecules31142437)
Supplement: Supplementary file 1 [file molecules-31-02437-s001.zip › Supplementary Table S4.pdf]

Table S4- Information about the studied vineyards

Vineyard	Granulometric composition	Humus, %	pH	Year of planting	Planting scheme	Vine-training system
Cabernet Sauvignon						
Vinogradny	Heavy loamy	1.0-1.9	7.15-8.11	2018	3.0 x 2.0	Cordon
Gostagaevskaya	Heavy loamy	1.2-3.2	7.20-7.70	2012	3.0 x 1.5	Cordon Double
Anapa	Medium loamy	0.6-1.9	7.45-8.00	2019	2.0 x 1.0	Guyot
Chardonnay						
Vinogradny	Light loamy	0.8-1.6	7.43-7.88	2018	3.0 x 2.0	Cordon
Gostagaevskaya	Heavy loamy	0.7-2.4	7.53-7.89	2019	3.0 x 1.5	Guyot
Anapa	Medium loamy	0.9-2.4	7.68-7.89	2013	3.0 x 1.5	Cordon Double
