# Supplementary material for: Rare Earth Elements in the Soil–Grape–Wine System: Opportunities and Limitations for Geographical Origin Authentication
Source: Molecules. 2026 Jul 11;31(14):2437. doi: 10.3390/molecules31142437 (PMC13415107; doi:10.3390/molecules31142437)
Supplement: Supplementary file 1 [file molecules-31-02437-s001.zip › Supplementary Table S5.pdf]

Table S5. Physico-chemical properties of Cabernet Sauvignon and Chardonnay wines

Wine	Indicator						
	Alcohol content, (%)	Titrated acidity, (g/L)	Volatile acids, (g/L)	Sugars, (g/L)	pH	Total phenols, (g/L)	SO_2,(mg/L)
Cabernet Sauvignon							
Anapa	12.1	4.8	0.60	2.4	3.8	2493	92
Vinogradny	9.1	10.8	0.53	1.5	3.3	1388	88
Gostagaevskaya	9.7	9.6	0.52	1.6	3.2	1458	78
Chardonnay							
Anapa	11.90	6.9	0.5	1.5	3.05	207	93
Vinogradny	11.60	6.6	0.48	0.3	3.14	189	75
Gostagaevskaya	11.80	6.6	0.54	1.3	3.14	144	86
