# Supplementary material for: Rare Earth Elements in the Soil–Grape–Wine System: Opportunities and Limitations for Geographical Origin Authentication
Source: Molecules. 2026 Jul 11;31(14):2437. doi: 10.3390/molecules31142437 (PMC13415107; doi:10.3390/molecules31142437)
Supplement: Supplementary file 1 [file molecules-31-02437-s001.zip › Supplementary Table S6.pdf]

Table S6 - Determination of REEs concentrations in CRM Soil (NCS DC 77302)

	Measured values			CRM Soil (avg ± sd)	Certified values with associated uncertainties	R, %
	Replicate 1	Replicate 2	Replicate 3			
La	38.2	36.9	38.0	37.7±0.57	37.6 ± 3.8	100.3
Ce	74.9	77.0	76.6	76.1±0.45	76.6 ± 8.4	99.4
Pr	8.71	8.71	8.67	8.69±0.02	8.8*	98.8
Nd	34.3	33.6	34.6	34.2±0.5	34.4 ± 3.4	99.4
Sm	6.69	6.51	6.75	6.65±0.12	6.6 ± 0.5	100.8
Eu	1.14	1.14	1.19	1.16±0.025	1.2 ± 0.2	96.7
Gd	5.66	5.71	5.55	5.64±0.08	5.6 ± 1.4	100.7
Tb	0.88	0.89	0.84	0.87±0.025	0.85 ± 0.28	102.4
Dy	5.33	5.29	5.30	5.31±0.01	5.3*	100.2
Ho	1.08	1.04	1.10	1.07±0.03	1.1*	97.3
Er	2.91	2.85	2.87	2.88±0.015	2.9*	99.3
Tm	0.45	0.47	0.52	0.48±0.026	0.48 ± 0.14	100.0
Yb	3.19	3.18	3.07	3.15±0.056	3.1 ± 0.2	101.6
Lu	0.46	0.47	0.48	0.47±0.0057	0.46 ± 0.12	102.2

* informative value
