# Supplementary material for: Rare Earth Elements in the Soil–Grape–Wine System: Opportunities and Limitations for Geographical Origin Authentication
Source: Molecules. 2026 Jul 11;31(14):2437. doi: 10.3390/molecules31142437 (PMC13415107; doi:10.3390/molecules31142437)
Supplement: Supplementary file 1 [file molecules-31-02437-s001.zip › Supplementary Table S7.pdf]

Table S7 - Determination of REEs concentrations in grapes

Element	Concentration, µg/L			
	Sample	Spike	Sample + Spike	R, %
La	0.89	1.00	1.96	103.8
	0.91	1.00	2.00	104.6
	0.92	1.00	2.02	105.4
Ce	1.86	1.00	2.78	97.1
	1.88	1.00	2.81	97.7
	1.81	1.00	2.85	101.4
Pr	0.24	0.20	0.45	102.0
	0.21	0.20	0.40	97.5
	0.22	0.20	0.44	104.0
Nd	1.21	1.00	2.15	97.1
	1.18	1.00	2.16	99.2
	1.17	1.00	2.18	100.3
Sm	0.22	0.20	0.44	105.0
	0.26	0.20	0.47	102.5
	0.23	0.20	0.41	96.3
Eu	0.12	0.10	0.22	99.2
	0.11	0.10	0.20	95.0
	0.12	0.10	0.22	99.2
Gd	0.21	0.20	0.41	99.0
	0.18	0.20	0.36	94.5
	0.20	0.20	0.38	95.9
Tb	0.041	0.40	0.417	94.6
	0.043	0.40	0.452	102.0
	0.039	0.40	0.422	96.2
Dy	0.15	0.20	0.36	103.0
	0.13	0.20	0.34	103.3
	0.14	0.20	0.35	102.4
Ho	0.029	0.20	0.23	101.6
	0.032	0.20	0.23	100.9
	0.033	0.20	0.23	97.8
Er	0.076	0.10	0.18	100.8
	0.078	0.10	0.17	95.9
	0.075	0.10	0.18	104.3
Tm	0.026	0.020	0.047	101.9
	0.028	0.020	0.046	95.3
	0.028	0.020	0.050	104.4
Yb	0.12	0.10	0.21	97.3
	0.11	0.10	0.20	95.4
	0.11	0.10	0.22	104.4
Lu	0.022	0.020	0.043	101.7
	0.021	0.020	0.040	97.2
	0.021	0.020	0.041	99.7
