# Supplementary material for: Rare Earth Elements in the Soil–Grape–Wine System: Opportunities and Limitations for Geographical Origin Authentication
Source: Molecules. 2026 Jul 11;31(14):2437. doi: 10.3390/molecules31142437 (PMC13415107; doi:10.3390/molecules31142437)
Supplement: Supplementary file 1 [file molecules-31-02437-s001.zip › Supplementary Table S8.pdf]

Table S8 - Determination of REEs concentrations in wines

Element	Concentration, μg/L			
	Sample	Spike	Sample + Spike	**R, %**
**La**	0.24	0.20	0.44	99.5
	0.25	0.20	0.46	102.4
	0.23	0.20	0.41	96.3
**Ce**	0.45	0.50	0.96	101.3
	0.49	0.50	0.95	96.2
	0.49	0.50	1.00	101.4
**Pr**	< 0.011	0.020	0.0198	98.9
	< 0.011	0.020	0.0198	99.1
	< 0.011	0.020	0.0191	95.4
**Nd**	< 0.014	0.020	0.0193	96.7
	< 0.014	0.020	0.0192	96.2
	< 0.014	0.020	0.0193	96.4
**Sm**	< 0.006	0.0050	0.0050	100.2
	< 0.006	0.0050	0.0049	98.5
	< 0.006	0.0050	0.0049	97.7
**Eu**	< 0.003	0.0050	0.0050	100.0
	< 0.003	0.0050	0.0052	104.5
	< 0.003	0.0050	0.0049	98.7
**Gd**	< 0.002	0.0050	0.0052	103.0
	< 0.002	0.0050	0.0049	97.4
	< 0.002	0.0050	0.0050	100.7
**Tb**	< 0.005	0.0050	0.0051	102.6
	< 0.005	0.0050	0.0051	102.2
	< 0.005	0.0050	0.0053	105.4
**Dy**	< 0.003	0.0050	0.0049	97.5
	< 0.003	0.0050	0.0052	104.7
	< 0.003	0.0050	0.0047	94.8
**Ho**	< 0.006	0.0050	0.0052	103.1
	< 0.006	0.0050	0.0050	99.1
	< 0.006	0.0050	0.0048	95.5
**Er**	< 0.003	0.0050	0.0052	103.3
	< 0.003	0.0050	0.0050	99.3
	< 0.003	0.0050	0.0051	101.5
**Tm**	< 0.002	0.0050	0.0047	94.6
	< 0.002	0.0050	0.0051	102.9
	< 0.002	0.0050	0.0049	97.7
**Yb**	< 0.002	0.0050	0.0048	95.9
	< 0.002	0.0050	0.0048	96.8
	< 0.002	0.0050	0.0049	97.7
**Lu**	< 0.002	0.0050	0.0048	95.9
	< 0.002	0.0050	0.0050	99.5
	< 0.002	0.0050	0.0050	100.2
