# Supplementary figures and images for: Rare Earth Elements in the Soil–Grape–Wine System: Opportunities and Limitations for Geographical Origin Authentication
Source: Molecules. 2026 Jul 11;31(14):2437. doi: 10.3390/molecules31142437 (PMC13415107; doi:10.3390/molecules31142437)

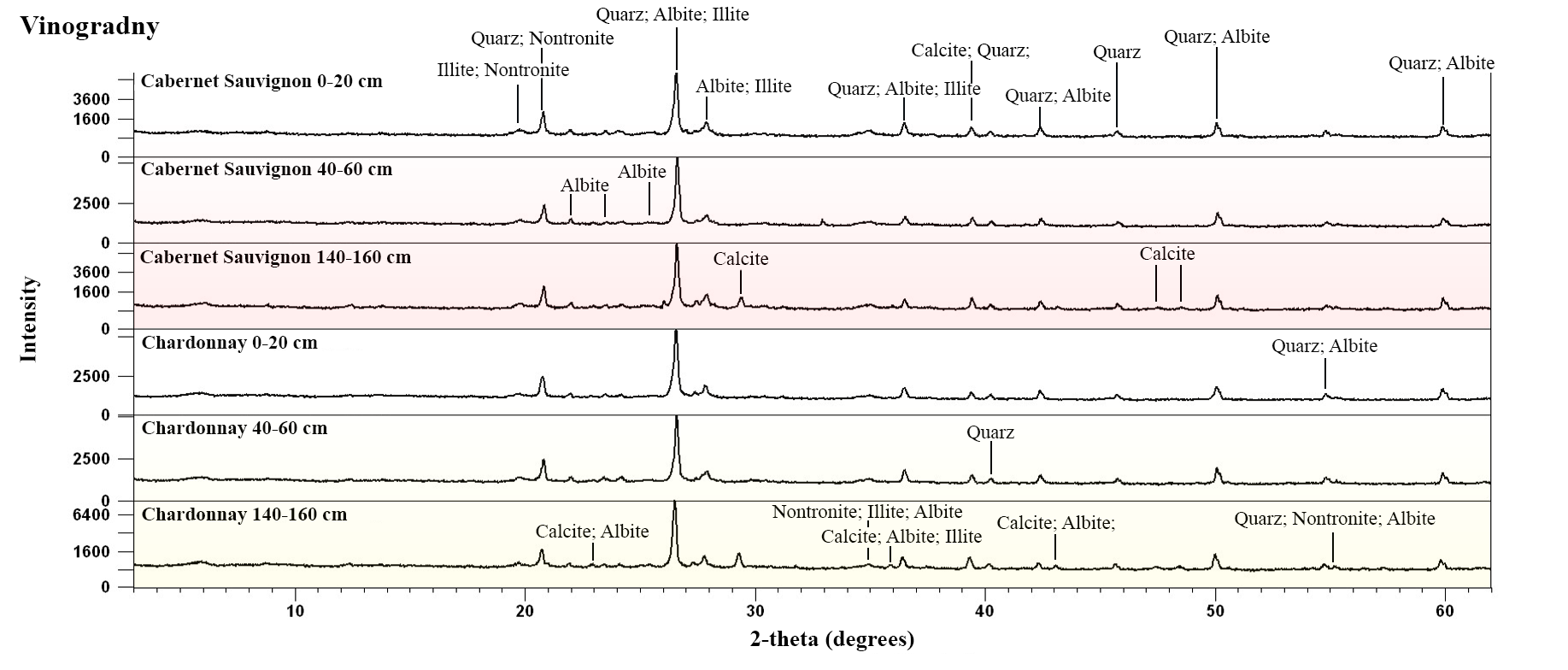

Supplement: Supplementary file 1 [file molecules-31-02437-s001.zip › Supplementary Figure S1.png]

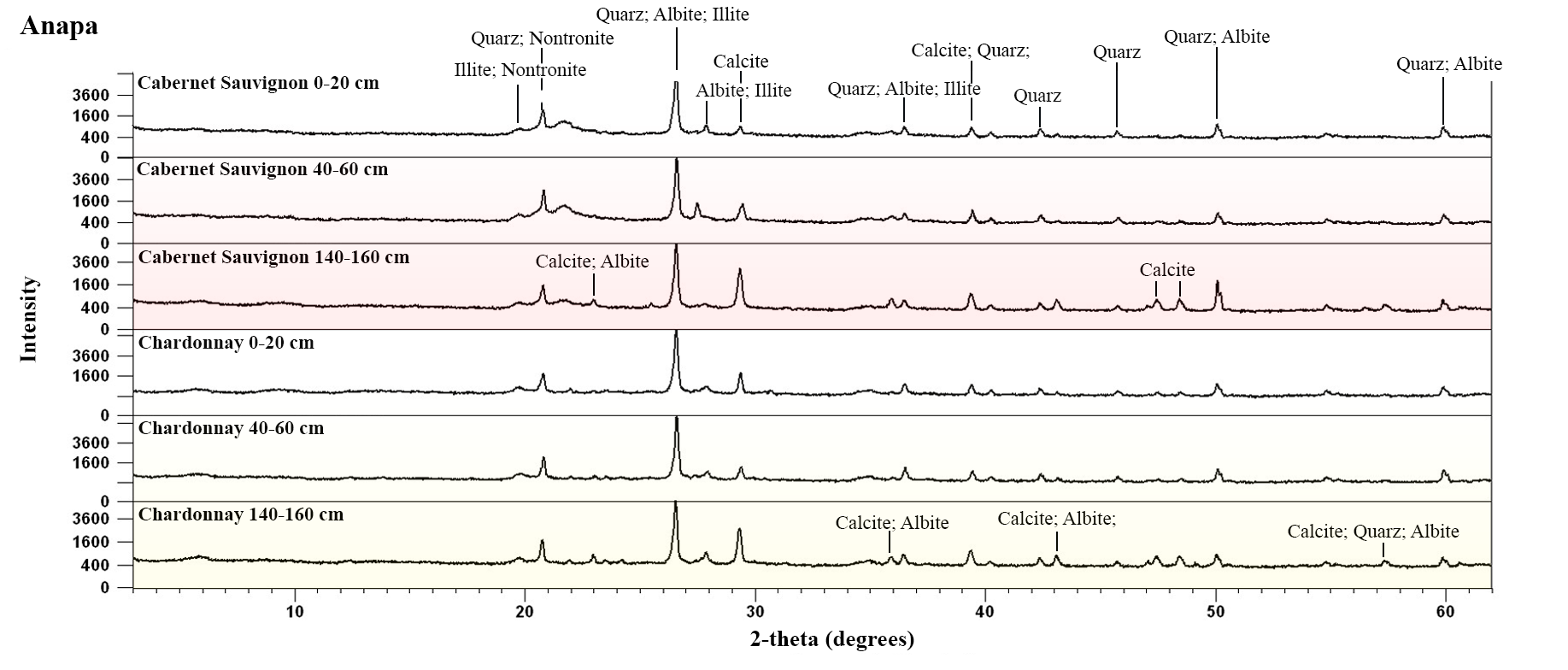

Supplement: Supplementary file 1 [file molecules-31-02437-s001.zip › Supplementary Figure S2.png]

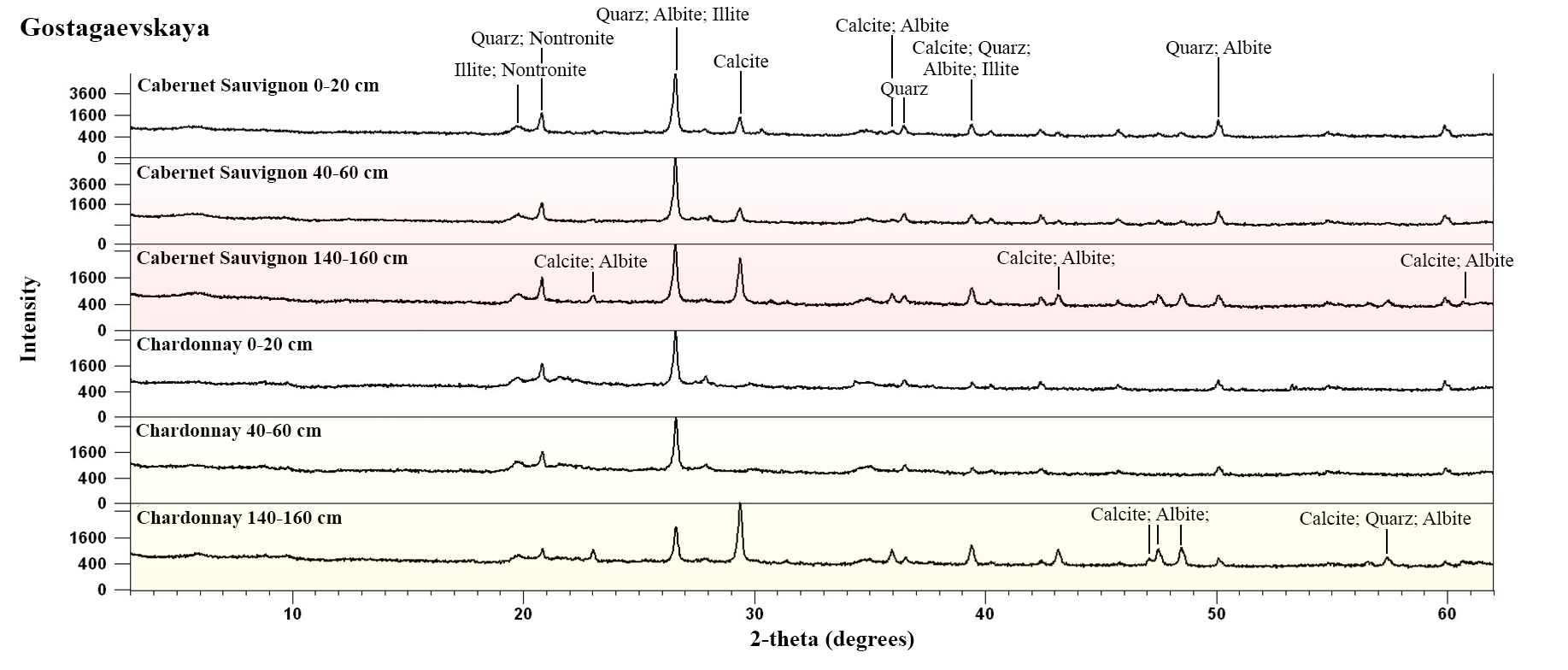

Supplement: Supplementary file 1 [file molecules-31-02437-s001.zip › Supplementary Figure S3.png]
